# The NeBoP score - a clinical prediction test for evaluation of children with Lyme Neuroborreliosis in Europe

**DOI:** 10.1186/s12887-015-0537-y

**Published:** 2015-12-17

**Authors:** Barbro H. Skogman, Johanna Sjöwall, Per-Eric Lindgren

**Affiliations:** Paediatric clinic, Falun General Hospital, Nissers väg 3, S-791 82 Falun, Sweden; Center for Clinical Research (CKF) Dalarna–Uppsala University, S-791 31 Falun, Sweden; Clinic of Infectious Diseases, Linköping University Hospital, S-581 85 Linköping, Sweden; Department of Clinical and Experimental Medicine, Medical Microbiology, Linköping University, S-581 85 Linköping, Sweden; Microbiological Laboratory, Medical Services, County Hospital Ryhov, S-551 85 Jönköping, Sweden

**Keywords:** Lyme neuroborreliosis, Lyme borreliosis, Predictive test, Diagnostic accuracy, Children

## Abstract

**Background:**

The diagnosis of Lyme neuroborreliosis (LNB) in Europe is based on clinical symptoms and laboratory data, such as pleocytosis and anti-*Borrelia* antibodies in serum and CSF according to guidelines. However, the decision to start antibiotic treatment on admission cannot be based on *Borrelia* serology since results are not available at the time of lumbar puncture. Therefore, an early prediction test would be useful in clinical practice. The aim of the study was to develop and evaluate a clinical prediction test for children with LNB in a relevant European setting.

**Method:**

Clinical and laboratory data were collected retrospectively from a cohort of children being evaluated for LNB in Southeast Sweden. A clinical neuroborreliosis prediction test, the NeBoP score, was designed to differentiate between a high and a low risk of having LNB. The NeBoP score was then prospectively validated in a cohort of children being evaluated for LNB in Central and Southeast Sweden (*n* = 190) and controls with other specific diagnoses (*n* = 49).

**Results:**

The sensitivity of the NeBoP score was 90 % (CI 95 %; 82–99 %) and the specificity was 90 % (CI 95 %; 85–96 %). Thus, the diagnostic accuracy (i.e. how the test correctly discriminates patients from controls) was 90 % and the area under the curve in a ROC analysis was 0.95. The positive predictive value (PPV) was 0.83 (CI 95 %; 0.75–0.93) and the negative predictive value (NPV) was 0.95 (CI 95 %; 0.90–0.99).

**Conclusion:**

The overall diagnostic performance of the NeBoP score is high (90 %) and the test is suggested to be useful for decision-making about early antibiotic treatment in children being evaluated for LNB in European Lyme endemic areas.

**Electronic supplementary material:**

The online version of this article (doi:10.1186/s12887-015-0537-y) contains supplementary material, which is available to authorized users.

## Background

Lyme Borreliosis (LB) is caused by the spirochete *Borrelia burgdorferi* and is the most common tick-borne infection both in Europe and Northern America [1, 2]. The infection may give rise to different symptoms by affecting organs such as the skin, joints, heart muscle or nervous system [3–5]. The diagnosis of Lyme neuroborreliosis (LNB) in Europe is based on clinical symptoms and laboratory findings, including pleocytosis in the cerebrospinal fluid (CSF) and intrathecal anti-*Borrelia* antibody production, in accordance with the guidelines [6]. However, the decision to start antibiotic treatment on admission cannot be based on anti-*Borrelia* antibodies in CSF, since test results are not available at the time of lumbar puncture. A prediction test would therefore be useful in clinical practice for decision-making about early start of antibiotic treatment.

Previous studies have suggested different clinical prediction rules but patients have not been representative of children with LNB in Europe [7–10]. Studies on large representative samples of all children being evaluated for LNB in European Lyme endemic areas are warranted.

Facial nerve palsy is the most common neurological finding among children with LNB in Europe [11, 12], but unspecific symptoms such as fatigue, low-grade fever, nausea and loss of appetite may often occur, without being accompanied with specific neurological findings [13]. The clinical picture in children with LNB is similar in Central and Northern Europe [14, 15] where the tick vector *Ixodes ricinus* is dominant and *Borrelia burgdoferi sensu lato* (*Bb*) is present in mainly three humanpathogenic species; *B. afzelii, B. garinii* and *Bb senso stricto* [1]. In Northern America, there are several different tick vectors and the main human pathogenic species is *Bb senso stricto* [2]. It is well known that the clinical picture of LNB in childhood differs in Europe compared to Northern America, where facial nerve palsy is less frequent but EM in combination with neurological symptoms occur more often [16–18]. Consequently, to find a common clinical predictive test valid for paediatric LNB patients on both continents is not feasible.

The aim of the study was to develop and evaluate a clinical prediction test for children with LNB in a relevant European setting.

## Methods

### Development of a clinical prediction test-the NeBoP score

Clinical and laboratory data was collected retrospectively from a large cohort of well-characterized and representative children being evaluated for LNB in Southeast Sweden during the period (2000–2005) (*n* = 177) [12]. This cohort was used for development and evaluation of the NeBoP score. Data was analysed in a logistic regression model to find independent and statistically significant variables to discriminate between “Confirmed LNB” and “Not determined”. Patients in “Confirmed LNB” were classified as “Definite LNB” patients according to European guidelines at the time [1], i.e. the same criteria as now [6]. Patients in “Not determined” were similar to “Non LNB”, i.e. patients with acute idiopathic facial nerve palsy, tension headache and patients with unspecific symptoms without LNB diagnosis [12]. Variables such as age, gender, headache, durations of symptoms, known tick bite or time of the year on admission did not differ between groups. Significant variables that came out in the logistic regression model were: 1) acute facial nerve palsy, 2) fever 38 - 39º C, 3) fatigue, 4) erythema migrans and/or lymphocytoma, 5) pleocytosis in CSF (with total cell count ≥ 5 × 10^6^/L with ≥ 90 % mononuclear cells).

**Fig. 1:**
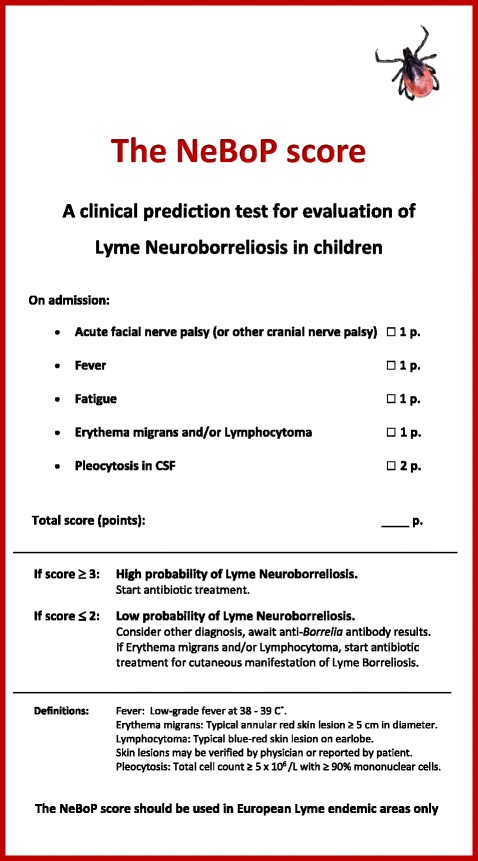
The NeBoP score, a clinical prediction test for children being evaluated for Lyme neuroborreliosis

Out of these five significant variables, the NeBoP score was designed, including weighed points (p) to differentiate between high and low probability of having LNB (Fig. 1). Definitions and instructions to the paediatrician were added to ensure equal and correct interpretation of the patient’s symptoms (Fig. 1). The NeBoP score was also pretested on a small group of paediatricians and minor corrections were made to ensure validity.

### Evaluation of cut-off levels for the NeBoP score

The performance of the NeBoP score at different cut-off levels was evaluated on data from the retrospective patient material [12] and shown in Table 1. A high sensitivity (100 %) at the best positive predictive value (0.60) was considered preferential, and the cut-off level was set at 3 points for a positive NeBoP score (Fig. 1 and Table 1).

**Table 1:** Cut-off levels for the NeBoP score

Cut-off	Sensitivity (%)	Specificity (%)	PPV ^#^	NPV ^*^
Score ≥ 2 p	100	42	0.54	1.00
Score ≥ 3 p	100	54	0.60	1.00
Score ≥ 4 p	78	64	0.60	0.82
Score ≥ 5 p	25	85	0.53	0.62

^#^ PPV = positive predictive value

* NPV = negative predictive value

p = score points (1–6 p)

Calculations are based on a retrospective cohort with “Confirmed LNB” as patients and “Not determined” as controls (12)

### Patient sample for validation of the NeBoP score

During the years 2010–2013, 197 children were evaluated for LNB at seven paediatric clinics in Central and Southeast Sweden. Children and parents/guardians were asked to participate in the study at the admission stage, and patients were consecutively enrolled as a prospective cohort. This cohort is considered representative for all paediatric patients being evaluated for LNB in a relevant European clinical setting, and is therefore suitable for this present study. CSF and blood samples were taken on admission for laboratory evaluation and the child and parents/guardians completed a standardized questionnaire. The paediatrician, following preset instructions and definitions, completed the NeBoP score for each patient on admission, before anti-*Borrelia* antibody results were available and before the patient was diagnosed as LNB or non-LNB. A two-month evaluation of clinical outcome was part of the study, but no serum samples were taken at follow-up.

Out of 197 patients included in the study, seven children were excluded because of missing data. These children (*n* = 7) did not differ in age or gender from patients included in the study (*n* = 190).

### Control sample for validation of the NeBoP score

Children being evaluated and diagnosed with other specific diagnoses during the study period were asked together with parents/guardians to participate in the study and were consecutively enrolled as controls (*n* = 49). Patients were not included from all seven paediatric clinics so this control sample cannot be considered as representative of children with each diagnosis. Instead, they represent a diversity of patients with other infectious, immunological and neurological diseases. Controls were children with enteroviral meningitis (*n* = 7), unspecified viral meningitis (*n* = 6), tick-borne encephalitis (*n* = 3), varicella zoster (*n* = 1), mycoplasma infection with neurological symptoms but negative PCR in CSF (*n* = 2), pneumonia with headache and normal CSF (*n* = 1), post-infectious encephalitis (*n* = 3), periodic fever (*n* = 1), unspecified autoimmune disease (*n* = 1), polyneuropathy (*n* = 1), Guillain-Barre syndrome (*n* = 1), multiple sclerosis (*n* = 1), myasthenia gravis (*n* = 1), narcolepsy (*n* = 1), neurofibromatosis type 1 (*n* = 1), ischemic stroke (*n* = 1), febrile seizure (*n* = 1), infantile spasm (*n* = 3), epilepsy (*n* = 2), idiopathic intracranial hypertension (*n* = 2), migraine headache (*n* = 3), tension headache (*n* = 3), head trauma (*n* = 3).

### Classification of patients and controls

According to European guidelines, classification of patients as “Definite LNB” and “Possible LNB” was based on neurological symptoms indicative for LNB and laboratory findings in CSF. [6] Patients who did not meet the criteria for either of the two groups were classified as “Non-LNB” and patients with other specific diagnoses were classified as “Controls” (Table 2).

**Table 2:** Classification of children being evaluated for Lyme neuroborreliosis and controls

Diagnosis	Criteria
Definite LNB ^§^	1. Neurological symptoms indicative for LNB without other obvious reasons
	2. Pleocytosis in CSF ^Ω^
	3. Intrathecal anti-*Borrelia* antibody production (IgG and/or IgM) ^#^
Possible LNB ^§^	Two of the criteria for Definite LNB are fullfilled
Non-LNB	Not meeting the criteria for Definite LNB or Possible LNB
Controls	Other specific diagnosis

^Ω^ Total cell count ≥ 5 x 10^6^/L in CSF

^§^ Classified according to European guidelines (6)

^#^ Detected by IDEIA Lyme neuroborreliosis assay (22)

LNB = Lyme neuroborreliosis

CSF = cerebrospinal fluid

Ig = Immunoglobulin

Pleocytosis in CSF was defined as total cell count ≥ 5 × 10^6^/L [19–21]. Intrathecal anti-*Borrelia* antibody production (IgG and/or IgM) was analysed with the routine assay IDEIA Lyme neuroborreliosis kit according to manufacturer’s instructions (Oxoid, Hampshire, UK) [22].

### Characteristics of patients

Clinical characteristics and laboratory data from patients being evaluated for LNB (*n* = 190) are shown in Table 3. Headache, fatigue, facial nerve palsy and loss of appetite were major clinical manifestations and known tick bite was reported from 53 % of patients. Ninety-nine patients (*n* = 99) received antibiotic treatment. Patients were diagnosed as “Definite LNB” (*n* = 52), “Possible LNB” (*n* = 31) and “Non-LNB” (*n* = 107) according to guidelines (Table 3) [6]. Patients in the “Non-LNB” were patients with acute idiopathic facial nerve palsy, tension headache and patients with unspecific symptom without LNB diagnosis.

**Table 3:** Characteristics of children being evaluated for Lyme neuroborreliosis

On admission	Patients (*n* = 190)
Age, median years (range)	10 (1–19)
Sex	
female, n (%)	105 (55)
male, n (%)	85 (45)
Known tick bite, n (%)	100 (53)
Major clinical features	
Acute facial nerve palsy, n (%)	93 (49)
Headache, n (%)	136 (72)
Fatigue, n (%)	144 (76)
Fever, n (%)	59 (31)
Neck pain, n (%)	71 (37)
Neck stiffness, n (%)	44 (23)
Loss of appetite, n (%)	89 (47)
Nausea, n (%)	68 (36)
Vertigo, n (%)	63 (33)
Radiating pain, n (%)	29 (15)
Erythema migrans (EM) and/or lymphocytoma, n (%)	42 (22)
Laboratory findings	
Pleocytosis in CSF, n (%) ^Ω^	82 (43)
with ≥ 90 % mononuclear cells, n (%)	75 (91)
Pleocytosis in CSF, median (range)	142 (8–890)
Anti-*Borrelia* antibodies in CSF, n (%) ^#^	53 (28)
IgM, n (%)	9 (5)
IgG, n (%)	12 (6)
IgM + Ig G, n (%)	32 (17)
Anti-*Borrelia* antibodies in serum, n (%)	83 (44)
IgM, n (%)	22 (12)
IgG, n (%)	26 (14)
IgM + Ig G, n (%)	35 (18)
Antibiotic treatment, n (%)	99 (52)
Diagnosis ^§^	
Definite LNB, n (%)	52 (27)
Possible LNB, n (%)	31 (16)
Non-LNB, n (%)	107 (56)

^Ω^ Total cell count ≥ 5 x 10^6^/L cells in CSF

^#^ Detected by IDEIA Lyme neuroborreliosis assay (22)

^§^ Classified according to European guidelines (6)

CSF = Cerebrospinal fluid

Ig = Immunoglobulin

LNB = Lyme neuroborrelios

### Characteristics of controls

Characteristics of children with other specific diagnoses (*n* = 49) are shown in Table 4. All controls were negative to anti-*Borrelia* antibodies in CSF but four children (*n* = 4) had anti-*Borrelia* IgG antibodies in serum. Known tick bites were reported from 24 % but no child in the control group had received antibiotic treatment for LNB. The different specific diagnoses among controls are described above under control sample.

**Table 4:** Characteristics of children with other specific diagnosis (controls)

On admission	Controls (*n* = 49)
Age, median years (range)	10 (0–19)
Sex	
Female, n (%)	26 (53)
Male, n (%)	23 (47)
Known tick bite, n (%)	12 (24)
Laboratory findings	
Pleocytosis in CSF, n (%) ^Ω^	15 (31)
with ≥ 90 % mononuclear cells, n (%)	4 (27)
Pleocytosis in CSF, median (range)	50 (6–1125)
Anti-*Borrelia* antibodies in CSF, n (%) ^#^	0 (0)
Anti-*Borrelia* antibodies in serum (IgG), n (%)	4 (8)
Diagnosis	
Viral meningitis (enterovirus), n (%)	7 (15)
Viral meningitis (unspecified), n (%)	6 (12)
Tick-borne encephalitis (TBE), n (%)	3 (6)
Other infectious disease, n (%)	4 (8)
Post-infectious encephalitis, n (%)	3 (6)
Other immunological disease, n (%)	2 (4)
Other neurological disease, n (%)	18 (37)
Tension headache, n (%)	3 (6)
Head trauma, n (%)	3 (6)

^Ω^ Total cell count ≥ 5 x 10^6^/L in CSF

^#^ Detected by IDEIA Lyme neuroborreliosis assay (22)

CSF = Cerebrospinal fluid

Ig Immunoglobulin

### Questionnaire

A structured questionnaire was used for data collection on admission and at the two-month follow-up. It consisted of questions to children and parents/guardians concerning current symptoms, known tick bites, previous antibiotic treatment of LB and the basic health of the child (as shown in Additional file 1). Children and parents/guardians in the control group received a similar, but slightly modified, questionnaire. At the two-month follow-up, questions focused on persistent symptoms and time to recovery.

### Statistics

**Fig. 2:**
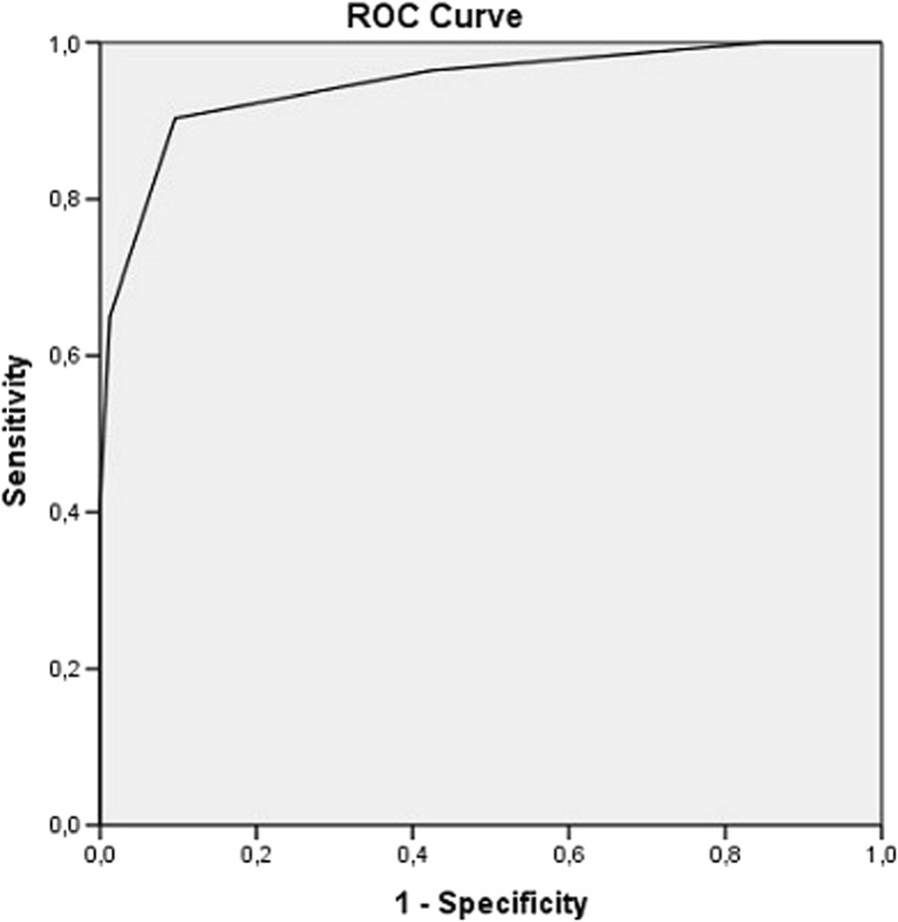
The diagnostic accuracy of the NeBoP score shown as a ROC curve. The area under the curve (AUC) was 0.95 (*p* < 0.0001). Calculations are based on “Definite LNB” (*n* = 52) and “Possible LNB”(*n* = 31) as patients and “Non-LNB”(*n* = 107) and “Controls”(*n* = 49) as controls. ROC curve = Receiver Operator Characteristic curve

SPSS software, version 21 and SISA-binomial were used for statistical calculations. A logistic regression was used to find independent and statistically significant variables to discriminate between “Definite LNB” and “Non LNB” in the retrospective cohort as described above. A *p*-value < 0.05 was considered significant. In the present prospective cohort, the diagnostic accuracy of the NeBoP score was calculated on “Definite LNB” and “Possible LNB” as patients and “Non-LNB” and “Controls” as controls. A receiver operating characteristic (ROC) curve with calculated area under the curve (AUC) was used to illustrate the results (Fig. 2).

### Ethics

Informed written consent was received from all children and parents/guardians included in the study. Approval of the study was obtained from the Regional Ethics Committee in Uppsala, Sweden (Dnr 2010/106).

## Results

### Diagnostic performance of the NeBoP score

Results from the NeBoP score in different diagnostic groups are shown in Table 5. Among children classified as “Definite LNB”, 51 out of 52 (98 %) had a positive NeBoP score and among children with “Possible LNB”, 24 out of 31 (77 %) had a positive test. The majority of children in “Non-LNB” and “Controls” had a negative NeBoP score (91 % and 90 % respectively) (Table 5).

**Table 5:** Results of the NeBoP score in different diagnostic groups

Diagnosis	NeBoP score		
	Positive	Negative	Total
Definite LNB, n (%)	51 (98)	1 (2)	52
Possible LNB, n (%)	24 (77)	7 (23)	31
Non-LNB, n (%)	10 (9)	97 (91)	107
Controls, n (%)	5 (10)	44 (90)	49

LNB = Lyme neuroborreliosis, classified according to European guidelines (6)

Positive test ≥ 3 points, negative test ≤ 2 points

The sensitivity of the NeBoP score was 90 % (95 % CI; 82–99 %), calculated on patients with “Definite LNB” and “Possible LNB”. The specificity of the test was 90 % (95 % CI; 85–96 %), calculated on patients with “Non-LNB” and “Controls” (Table 6). Thus, the overall diagnostic accuracy of the NeBoP score (i.e. how the test correctly defines patients and controls) was 90 %. Results are also shown as a ROC curve with an area under the curve (AUC) of 0.95 (*p* < 0.001) (Fig. 2).

**Table 6:** Diagnostic performance of the NeBoP score

	NeBoP score
Sensitivity, (95 % CI)	90 % (82–99 %)
Specificity, (95 % CI)	90 % (85–96 %)
Positive predictive value (PPV), (95 % CI)	0,83 (0.75–0.93)
Negative predictive value (NPV), (95 % CI)	0,95 (0.90–0.99)
Positive likelihood ratio (LR+), (95 % CI)	9.34 (5.05–17.47)
Negative likelihood ratio (LR-), (95 % CI)	0.11 (0.05–0.25)

Calculations are based on “Definite LNB” (*n* = 52) and “Possible LNB”(*n* = 31) as patients and “Non-LNB” (*n* = 107) and “Controls”(*n* = 49) as controls

CI = Confidence interval

The positive predictive value (PPV) of the NeBoP score was 0.83 (95 % CI; 0.75–0.93) and the negative predictive value (NPV) was 0.95 (95 % CI; 0.90–0.99). Likelihood ratios (LR) are shown in Table 6.

### Distribution of clinical symptoms and NeBoP score points

**Fig. 3:**
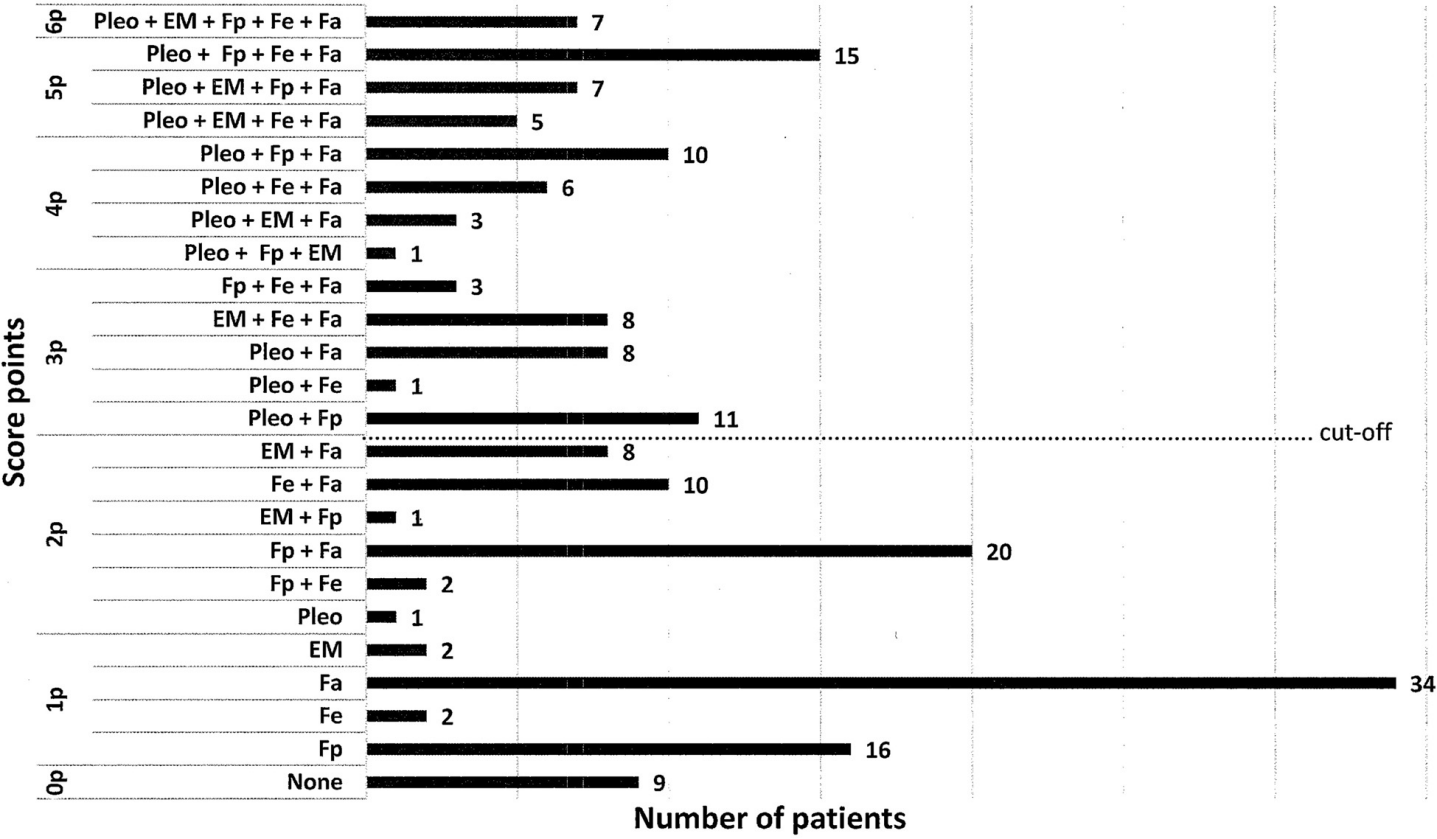
Distribution of clinical symptoms and NeBoP score points among children being evaluated for Lyme neuroborreliosis (*n* = 190). Pleo = pleocytosis (total cell count ≥ 5 x 10^6^/L cells in CSF with ≥ 90 % mononuclear cells), EM = erythema migrans, Fp = facial nerve palsy, Fe = fever, Fa = fatigue, NeBoP = Neuroborreliosis Prediction, p = score points with cut-off ≥ 3 p for a positive test

The distribution of clinical symptoms and NeBoP score points among children being evaluated for LNB (*n* = 190) are shown in Fig. 3. Among patients with ≥ 3 p in the NeBoP score, the most common combination of symptoms was facial nerve palsy, low-grade fever and fatigue in combination with pleocytosis in CSF (Fig. 3).

## Discussion

This study shows a high diagnostic accuracy (90 %) of the NeBoP score in children being evaluated for LNB in a Northern European Lyme endemic area. Consequently, our results support that this clinical predictive test could be useful for paediatricians in early assessment of children being evaluated for LNB. The NeBoP score is applicable in Lyme endemic areas in Central and Northern Europe since there are known similarities in LNB in childhood [15, 16]. However, the test is not recommended for Northern America due to differences in the clinical manifestations of LNB, the occurrence of different tick vectors and different *Borrelia* species between the two continents [16, 17].

Admittedly, there are a few patients being incorrectly predicted with using the NeBoP score in the present study, which needs to be addressed. Among children classified as “Definite LNB”, 98 % had a positive NeBoP score (≥3 p) but one patient had negative test (Table 5). This patient had unilateral abducens palsy with pleocytosis and anti-*Borrelia* antibodies in CSF, (correctly classified as “Definite LNB”) but received only 2 NeBoP score points (negative test). Consequently, the NeBoP score should also include other cranial nerve palsy since LNB patients may present with both abducens-and/or oculomotorius nerve palsy [23]. This detail is added in the final version of the NeBoP score (Fig. 1).

Among children classified as “Possible LNB”, the majority (77 %) had positive NeBoP scores whereas seven patients (23 %) had negative tests (Table 5). Six of these patients presented with short duration of symptoms (1–6 days of headache and/or facial nerve palsy) in combination with pleocytosis in CSF, indicating an early LNB. One patient had anti-*Borrelia* IgM antibodies in serum, one had anti-*Borrelia* IgG antibodies and one had both IgM and anti-*Borrelia* IgG antibodies in serum. All patients responded well to antibiotic treatment. It is uncertain whether these six patients were LNB patients with negative NeBoP score due to an untypical distribution of percentage of mononuclear cells in CSF (40–88 % of total cell count in CSF) or whether symptoms actually derive from other etiology. Unfortunately, these patients were not tested for other tick-borne infections or enteroviral PCR in CSF. Furthermore, one patient with a negative NeBoP score in the “Possible LNB” group presented with very long duration of symptoms, anti-*Borrelia* IgG antibodies in CSF but no pleocytosis in CSF. The patient had a history of a previously treated LNB. Findings may indicate persistent symptoms as sequelae after a previous LNB infection (despite adequate antibiotic treatment) or ongoing infection without pleocytosis in CSF. The patient again received antibiotic treatment and symptoms were slightly reduced but did not resolve totally.

Our results show that the sensitivity of the NeBoP score is excellent in “Definite LNB”, (98 %) and acceptable in “Possible LNB” (77 %), with an overall sensitivity of 90 % for the two groups together. Thus, the NeBoP score will help the paediatrician to decide about early antibiotic treatment before test results of anti-*Borrelia* antibodies in serum and CSF are available. Regarding the *Borrelia* serology, one should always keep in mind that both anti-*Borrelia* IgG and IgM antibodies in serum should be interpreted with caution because of low sensitivity and specificity [6].

Concerning the two control groups in this study, the heterogeneity of the negative controls (*n* = 49) could be put under further considerations since they represent many different diagnoses without similar clinical manifestations to LNB. However, when evaluating a predictive test it is of importance to include controls *without* clinical similarity to patients (49 negative controls) as well as controls *with* clinical similarity to LNB patients from a clinical relevant setting (107 Non-LNB patients). We have included these two control groups in our study and we believe the heterogeneity of controls with other diagnosis therefore can be acceptable. Negative controls *without any* symptoms (i.e. healthy controls) could not be included in the study due to the fact that a lumbar puncture cannot be performed on healthy children out of ethical reasons.

A control group including a higher number of patients with enteroviral and/or bacterial meningitis should admittedly have been preferable, which is a weakness of the study. Furthermore, the sample size of negative controls could have been larger than 49, but it could unfortunately not be achieve during the time period and in clinical setting of the study. Median age and sex distribution do not differ between patients (Table 3) and controls (Table 4), which is a strength of the study.

In the “Non-LNB” group, 91 % had a negative NeBoP score (≤2 p) indicating a high specificity (Table 5). However, 10 children (9 %) in “Non-LNB” scored ≥3 p (positive test). Three of these 10 children had acute facial nerve palsy, fever and fatigue with short duration of symptoms, but no pleocytosis or anti-*Borrelia* antibodies in CSF. These patients may have had idiopathic facial nerve palsy or very early LNB with peripheral cranial nerve palsy but not yet pleocytosis in CSF. Furthermore, seven patients in the “Non LNB” group scored ≥3 p (positive test) due to fever, fatigue and EM but had normal CSF, indicating a cutaneous LB with systemic symptoms. These patients are probably incorrectly predicted as LNB by the NeBoP score and should instead be classified as cutaneous LB and receive treatment as such. This instruction is added in the final version of the NeBoP score (Fig. 1). Again, it should be pointed out how important an evaluation of CSF is, concerning patients with EM and systemic symptoms, since symptoms may indicate an early LNB [24].

Among controls, four children scored ≥3 p (positive test) due to viral meningitis with fatigue and pleocytosis with ≥ 90 % mononuclear cells in CSF, yielding a false positive NeBoP score. However, these patients had a clinical picture with clear meningeal symptoms in addition to fever and fatigue, making it easy for the paediatrician to clinically distinguish them from patients with Lyme meningitis. Two of them were positive for enterovirus PCR in CSF.

One patient among controls had a periodic fever with fatigue, low-grade fever and a red skin lesion similar to an EM, which resulted in a misclassification by the NeBoP score (3 p). EM is a clinical diagnosis but it is well know that the EM skin lesions may be heterogeneous and may result in misdiagnosis [24]. In such cases, *Borrelia* serology is not useful due to low sensitivity [24].

In our cohort of children, 15 out of 107 patients in “Non-LNB” received antibiotic treatment on admission before anti-*Borrelia* antibody results were available. We believe, based on results from our present study with a NPV of 0.95 for the NeBoP score, that the test will be helpful for the paediatrician to correctly refrain from antibiotic treatment on admission and consider other differential diagnoses while waiting for anti-*Borrelia* antibody results.

Whether the NeBoP score is more helpful as a decision tool for the paediatrician in the early assessment of children being evaluated for LNB as compared to the pleocytosis itself, as a single variable, can be discussed. It has previously been shown that pleocytosis with ≥ 90 % mononuclear cells in CSF clearly discriminates Lyme meningitis from viral meningitis [19, 25–28]. However, there are patients without pleocytosis in CSF with cranial nerve palsy, fatigue and fever who will be detected by a positive NeBoP score and be recommended early start of antibiotic treatment. Furthermore, it has been shown that the majority of symptoms in children with LNB resolve within a few days after the start of antibiotic therapy [29], but whether an early start of treatment is favorable for long term clinical outcome is not clear [30].

A prediction model for LNB in children in a European setting has, to our knowledge, previously been presented only in one study [25]. However, children with acute facial nerve palsy without meningitis were not included, which qualifies our NeBoP score as a more relevant predictive test with a more adequate representation of all children being evaluated for LNB in a relevant European setting. Furthermore, a clinical prediction test would also be useful for adult LNB patients, but such a test has, to our knowledge, not yet been developed.

## Conclusion

In conclusion, the overall diagnostic accuracy of the NeBoP score is high (90 %) and the test is suggested to be useful for decision-making about early antibiotic treatment in children being evaluated for LNB in European Lyme endemic areas.

## Additional file

Additional file 1:
**Questionnaire.** Study ”Lyme Neuroborreliosis in children”. (DOC 2013 kb)

## Abbreviations

AUC: Area under the curve
*Bb*: *Borrelia burgdorferi*
CI: Confidence interval
CSF: Cerebrospinal fluid
EM: Erythema migrans
IgG: Immunoglobulin G
IgM: Immunoglobulin M
LB: Lyme borreliosis
LNB: Lyme neuroborreliosis
LR: Likelihood ratio
NeBoP score: Neuroborreliosis prediction score
NPV: Negative predictive value
PCR: Polymerase chain reaction
PPV: Positive predictive value
ROC curve: Receiver operator characteristic curve
