# Supplementary material for: The NeBoP score - a clinical prediction test for evaluation of children with Lyme Neuroborreliosis in Europe
Source: BMC Pediatr. 2015 Dec 17;15:214. doi: 10.1186/s12887-015-0537-y (PMC4682231; doi:10.1186/s12887-015-0537-y)
Supplement: Additional file 1: — Questionnaire. Study ”Lyme Neuroborreliosis in children”. (DOC 2013 kb) [file 12887_2015_537_MOESM1_ESM.doc]

##
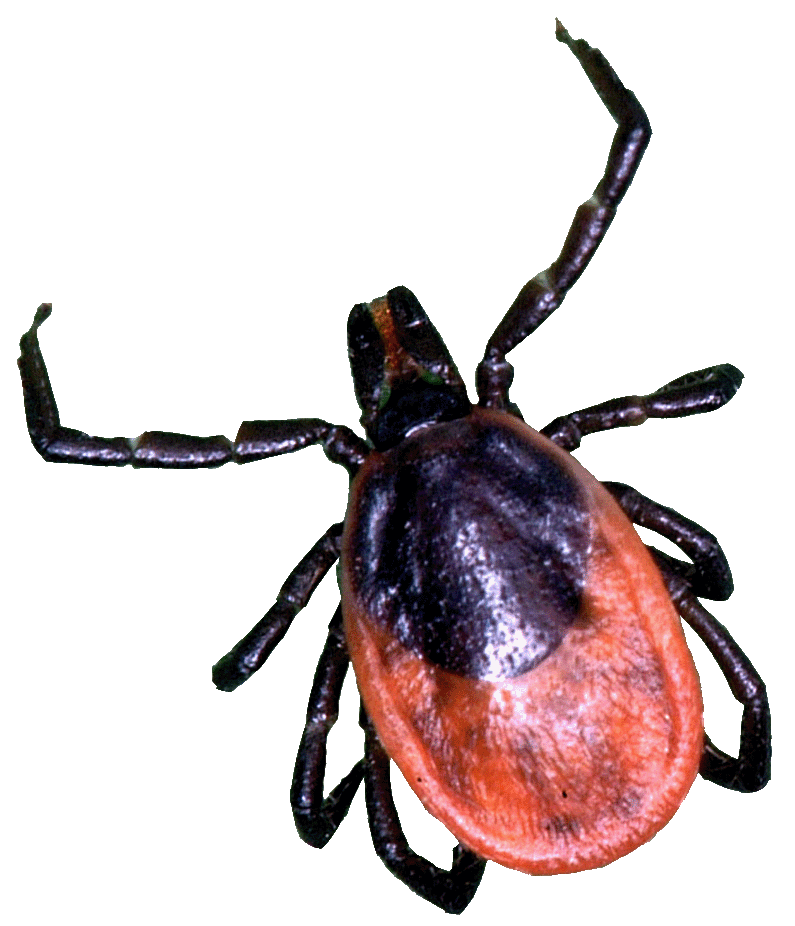
Study ”Lyme Neuroborreliosis in children”

**Questionnaire**  (by parents and/or guardians) Code:________________________

Name:_________________________________________Hospital:_____________________

Date of birth: ___________________________________Date for inclusion:______________

*****************************************************************************Questions:**

**YES NO** If yes, duration of symptoms at admission:

1-2 d 3-6 d 1-2 w 2-4 w 1-2 m >2 m

1. Facial nerve palsy:

2. Headache:

3. Fatigue:

4. Fever (38-39^0^ celcius):

5. Neck pain:

6. Neck stiffness:

7. Loss of appetite:

8. Nausea/vomiting:

9. Vertigo:

10. Radiating pain

11. Other symptom:

(if yes, what symptom:............................................................................................................. .....................................................................................................................................................)

**YES NO** If yes, duration at admission:

1-4 w 1-2 m 3-5 m 6-12 m >1 y

12. Tick bite:

(if yes, where?.....................................................................................................................

13. Red skin lesion (erythema migrans):

(if yes, where?......................................................................................................................

14. Swollen earlobe (lymphocytoma):

15. Vesicles on lip (herpes simplex):

16. Vesicles on skin (varicella zoster):

17. Vaccination for TBE:

18. Vaccination for Yellow fever:

***************************************************************************

19. Treatment for previous *Borrelia* infection (drug, dose)?…………………………………..

……………………………………………………...…….……………………………………

20. Where in Sweden has the child been spending summer vacation the last two years?

…………………………………………………………………………………………...............

21. Is the child healthy (yes/no)?..................................................................................................

22. If no, what is the problem (diagnosis)?...................................................................................

....................................................................................................................................................... 23. Treatment?..............................................................................................................................
